# Alternatives to Gold Standard Diagnostic Tools for Distinguishing “Natural Kinds” on the Autism Spectrum

**DOI:** 10.3389/fpsyt.2022.862410

**Published:** 2022-06-06

**Authors:** Anne Philippe

**Affiliations:** Developmental Brain Disorders Laboratory, INSERM UMR 1163, Imagine Institute, Université Paris Cité, Paris, France

**Keywords:** autism spectrum disorder, rare single-nucleotide variants, rare copy-number variants, psychopathological phenomenon, Asperger, Newson, Wing, Falret

## Abstract

Next-generation sequencing techniques have accelerated the discovery of rare mutations responsible for autism spectrum disorder (ASD) in genes involved in a large number of physiological processes, including the control of gene expression, chromatin remodeling, signaling pathways, synaptic scaffolding, neurotransmitter receptors, and lipid metabolism. Genetic diagnosis provides subjects with an explanation of the cause of their disorder. However, it does not, or at least does not yet, shed light on the psychopathological phenomena specific to the individual. It could be hypothesized that each physiological impact of a mutation corresponds to a specific psychopathological phenomenon of ASD, i.e., “a psychopathological natural kind”. We discuss here the difficulties identifying this specificity of underlying psychopathology in individuals with ASD due to a rare mutation with a major effect. A comparison of Newson's *pathological demand avoidance* and Wing's *Asperger's syndrome* with Asperger's *autistic psychopathy* highlights different ways of approaching psychopathological descriptions and diagnosis, by focusing on either common or unusual features. Such a comparison calls into question the principles of clinical research recommended by Falret for characterizing “disease individuality” of ASD due to a rare mutation.

## Introduction

Autism spectrum disorder (ASD) is a heterogeneous condition, with many etiologies, characterized by impaired social communication, repetitive behaviors, and highly restricted interests and/or sensory behaviors beginning early in life (1).

In the era of next-generation sequencing, the use of a *genotype-first* approach combined with *reverse phenotyping* has led to the identification of pathogenic genetic variants underlying ASD in more than a hundred genes over the last decade. Pathogenic variants are individually rare, but collectively, they allow genetic diagnosis in 10–30% of individuals with ASD (2).

This strategy involves grouping individuals with a *rare* potentially damaging variant of the same gene together (genotype-first step) and then comparing their phenotypes *a posteriori* (reverse phenotyping step), in greater detail, to determine whether the individuals in the same group share the same specific phenotype (2, 3). If the *specific* phenotype appears to be consistent in at least three unrelated individuals, variants of this gene can be considered pathogenic, allowing the description of a novel “natural kind” (4).

This recognition of a *specific* phenotype is facilitated when the cognitive affective behavioral disturbances are associated with specific somatic or paraclinical signs, such as dysmorphic facial features (5), macrocephaly (6), or abnormal cerebellar foliation (7). In these cases, ASD is considered syndromic (8).

In the absence of objective signs (non-syndromic ASD), the recognition of a specific cognitive affective behavioral phenotype is more difficult, requiring intersubjective methods, such as observation, scales and questionnaires, interviews with the individual and their relatives, or psychological tests, resulting in a greater divergence of points of view between clinicians.

In this article, we discuss how best to investigate the specific psychopathology of ASD subjects with rare pathogenic variants, assuming that each physiological impact of a mutation corresponds to a specific psychopathology.

### Elizabeth Newson's *Pathological Demand Avoidance Syndrome*

Identification of the specific “psychopathological natural kind” of ASD subjects with a rare pathogenic variant requires an extensive knowledge of normal child development and of the various clinical entities described within and beyond ASD, such as *autism* (9, 10), *schizoid/autistic psychopathy* (11, 12), *multiple complex developmental disorder* (13), and *schizoid personality* (14), neuropsychological syndromes, such as *nonverbal learning disabilities syndrome* (15), *semantic-pragmatic disorder* (16), *developmental prosopagnosia* (17), *cerebellar cognitive affective syndrome* (18), and *executive dysfunction* (19), and differential diagnoses, such as *Landau-Kleffner syndrome* (20), *early-onset schizophrenia* (21), *early-onset catatonias* (22), and *attachment disorders* (23).

It is within this context that we are discussing the clinical entity proposed by Elizabeth Newson, “pathological demand avoidance syndrome”, which she described as a necessary distinction within the pervasive developmental disorders, but which is little known outside of Great Britain (24).

In the 1980s Elizabeth Newson (1929–2014), a professor of developmental psychology at the University of Nottingham, described a particular behavioral pattern in a subgroup of children some of whom had been referred to her for suspected autism or Asperger's syndrome. However, these diagnoses were not confirmed by her own diagnostic assessment, and she considered these children to have what she called “pathological demand avoidance” (PDA) (24).

The key characteristics of these children were: (1) feeling intolerable pressure when faced with the most ordinary of everyday demands, and using various manipulative strategies to avoid most of the requests they encountered, (2) a superficial sociability that was invested in a self-centered manner, (3) mood swings or impulsivity in response to a request or for no apparent reason, and (4) a marked interest in an imaginary world.

Newson showed PDA to be different from both classical autism and Asperger's syndrome in a functional analysis on 90 children (50 with PDA, 20 with classical autism, 20 with Asperger's syndrome). She considered PDA to be a *specific* pervasive developmental disorder (24). However, the validity of her results was limited by several methodological flaws: the diagnostic criteria for the three groups compared were not defined, Autism Diagnostic Interview-Revised (ADI-R) testing was not used to ensure that none of the children with PDA also met the criteria for ASD, etc.

### Close to Hans Asperger's *Autistic Psychopathy*?

Given the seeming convergence, it is odd that Newson did not draw parallels between her syndrome and that of the four autistic psychopathy cases, Fritz, Harro, Ernst and Helmut described by Asperger (11). Indeed, as described by Asperger:

“from the earliest age Fritz never did what he was told. He did just what he wanted to, or the opposite of what he was told. He was utterly indifferent to the authority of adults, he called everybody ‘Du'. As one would expect, the conduct disorders were particularly gross when demands were made on him. In fact, it is typical of children such as Fritz that they do not comply with requests or orders that are affectively charged with anger, kindness, persuasion or flattery. Instead, they respond with negativistic, naughty and aggressive behavior” (11).

Harro presented similar discipline problems: “[He] often refused to co-operate, sometimes using bad language. He rarely did what he was told but answered back and with such cheek that the teacher had given up asking him. He was said to be an inveterate ‘liar'. He did not lie in order to get out of something that he had done - this was certainly not the problem, as he always told the truth very brazenly - but he told long, fantastic stories” (11).

The article “*Die Austitischen Psychopathen im Kindesalter*” (1944) remained almost unknown for many years, probably because it was originally written in German. An unabridged English translation did not become available until 1991 (11), so Newson may have been unaware of it when she developed the PDA concept in the 1980s. In any case, she did not mention it in the references of her 2003 article (24).

### Different From Lorna Wing's *Asperger's Syndrome*?

Lorna Wing (1928–2014) introduced Hans Asperger's article to the scientific community in an article published in 1981, entitled “Asperger's syndrome: a clinical account”, in which she redefined the syndrome starting from the original description, but with alterations drawn from her own experience. Indeed, Wing disagreed with Asperger, disputing the “highly sophisticated linguistic skills” of these children and any creativity and originality that they may have displayed, instead focusing on their absence of social imagination (25).

Her search for severe impairments of social interaction and repetitive stereotyped behaviors, initially in disabled children and then among children without intellectual deficiency, revealed that these children shared many abnormalities. This led her to propose a “continuum” between Kanner's autism and other related entities, such as Asperger's autistic psychopathy or PDA (26, 27), which greatly influenced the current concept of “autism.” This view diverged from former representations, such as Folstein and Rutter's first twins' study (1977), in which they regarded a pair of monozygotic twins as discordant because one presented autistic disorder and the other, Asperger's autistic psychopathy (28). Wing also contributed to the introduction of Asperger's syndrome into the DSM-IV. Nevertheless, the difference between Asperger's original description and the definition in the DSM-IV generated confusion among clinicians. Asperger's syndrome was, therefore, removed from the DSM-5, instead included, though a dimensional approach, in a diagnostic classification within a new category -autism spectrum disorder- along with autism and pervasive developmental disorder-not otherwise specified.

So, were Newson and Wing talking about the same children? Do these children display clinical variability or clinical heterogeneity? Are their *socio-communicative deficits* and *pathological demand avoidance* two facets of the same disorder?

To answer these questions, we encourage clinicians to read the translation of the original article by Asperger (“Die autistischen Psychopathen im Kindesalter”), from the book by Uta Frith (11), in its entirety so that they can judge for themselves whether the Asperger's original description is identical to or different from PDA and Wing's Asperger's syndrome.

## Discussion

The difficulty of directly accessing the mental state underlying the signs and symptoms of a subject makes it difficult to develop nosographies in psychiatry.

The DSM and its successive versions marked a turning point in the history of psychiatry, by proposing a classification widely used in applied research for constituting groups of patients. This has improved inter-rater agreement, and, thus, the reliability of diagnosis, with the use of a common language facilitating exchanges in clinical practice (29). There occasionally are disagreements between clinicians, as illustrated by the case report of “An 8-year-old boy with school difficulties and “odd behavior” (30). The behavior of this boy suggested a diagnosis of Asperger's syndrome (“happy to play alone”) but his teacher found “[his] social skills were a notable strength”, which led to several other potential diagnoses, such as ADHD and obsessive-compulsive disorder, and diverse suggestions for therapeutic strategies ranging from “wait and see” to treatment with methylphenidate (30).

In basic research, the validity of the DSM has been called into question (31), including for ASD (32), due to a lack of knowledge of the underlying etiopathological mechanisms.

The identification, in recent years, of rare pathogenic variants [single-nucleotide variants (SNVs) or copy-number variants (CNVs)] in individuals with ASD has made it possible to develop a taxonomy, based on an etiological approach (33). Each type of physiological impact of a mutation, resulting, for example, in a decrease or increase in gene expression or a loss-of-function or a gain-of-function in protein, may correspond to a specific psychopathological form of ASD that can be seen as a “psychopathological natural kind”.

These discoveries provided some initial insight into physiological mechanisms, through explorations of the impact of these pathogenic variants in functional studies *in vitro* and *in vivo* (34). They made it possible to group subjects with the same rare pathogenic genomic abnormality together, in a way that current clinical assessment cannot, either because clinical manifestations are not very specific or because they differ too much between subjects according to contemporary classifications. This grouping together of small numbers of subjects makes it possible to evaluate the *principle of specificity* defended by Bretonneau in the field of infectious diseases at the start of the nineteenth century, by assessing the correspondence between genetic and psychopathological diagnoses (35).

This principle of specificity often initially appears not to be respected in ASD, particularly in non-syndromic forms, whether for SNVs or CNVs. Partial deletions of the *SHANK3* gene, for example, can manifest in ASD subjects with and without intellectual deficiencies (36), and proximal 16p11.2 duplication (BP4–BP5) may arise *de novo* in an ASD subject or be inherited from a “healthy” parent, highlighting the incomplete penetrance and/or variable expressivity (37).

Before studying the influence of background factors, including “concrete” variables, such as environmental factors (exposure *in utero* to toxic substances, neonatal distress, psychosocial environment), epigenetic and stochastic factors (monoallelic or allele-biased gene expression), and genetic background, on *phenotypic heterogeneity* in subjects carrying a given pathogenic variant, it is important to describe the *phenotypic variability* of the “natural kind” associated with the variant in question.

From a fundamental research perspective, ASD lies at the frontier between life sciences, supported by a naturalist framework for studying objects with a “concrete” reality (signs, mutations, dysembryoplastic neuroepithelial tumor, tubers, etc.), and human sciences, which function within a normative framework, exploring complex objects often constructed in a more abstract manner (symptoms, idiosyncratic interests, psychiatric diagnosis, etc.) (38).

Improvements in our knowledge of ASD require continual switching between these two types of science. For example, studies of the expression of mutated genes have improved our understanding of the reasons for which only a subgroup of patients suffering from neuromuscular diseases (e.g., the Duchenne and Becker dystrophies linked to the *DMD* gene, dyneinopathies linked to the *DYNC1H1* gene) present cognitive, emotional, and/or behavioral problems (including ASD). Such studies have identified mutations located in particular domains of the gene that lead to specific physiological alterations to the resulting transcripts in the brain (e.g., the Dp71 transcript of the *DMD* gene) (39, 40).

The *reverse phenotyping* approach has drawn attention to certain behavioral peculiarities, such as obsessive-compulsive disorder in Prader-Willi syndrome and social anxiety disorder in fragile X syndrome (41). However, the nature of these disorders remains to be understood, and a methodological reflection is required to describe the underlying psychopathology of “natural kinds.” Indeed, it is not a question of confirming or rejecting a known psychiatric diagnosis, but of identifying psychopathological consequences with, *a priori*, signs that are new or have rarely been taken into account.

The DSM is not particularly suitable for identifying the unusual clinical manifestations corresponding to rare mutations. This tool is based on the findings of epidemiological studies on large samples, which favor frequently occurring symptoms over rare, often more specific, signs or symptoms. It consists of diagnostic categories defined by the presence of sufficient symptoms from a specific list, but it does not take the context into account, which is essential to understand the psychopathological value of the manifestations. Finally, for therapeutic purposes, the DSM concerns only “clinically significant” manifestations, whereas basic research is equally interested in the “silent side of the spectrum” (42, 43).

In addition, the DSM, with its operational criteria defining each clinical category, has profoundly modified the psychiatric semiological approach of an entire generation of clinicians, by structuring the representation of disorders around these criteria, which are sometimes abusively considered to be exhaustive (44). The recognition of signs and symptoms is, thus, strongly influenced by the category of the suspected diagnosis, and this may constitute an epistemological obstacle to the psychiatric observation of unusual clinical manifestations (45).

So, how can we discover the “psychopathological natural kind”, given that “what is observed is often neither relevant nor significant, and what is relevant and significant is often difficult to observe” (46)?

We can now return to the starting point, the principles recorded by Falret in his *Clinical Lessons* in 1864, which enabled him to identify “circular insanity” (1854), now known as bipolar disorder, which he considered, along with general paralysis, to be a model of “natural forms” (47–49).

The *first principle* is “do not reduce your responsibility as an observer to the passive role of the patient's secretary” (47, 48). Careful clinical observation is the cornerstone of the clinical method, but clinicians must also play an active role in “bringing out manifestations that would not arise spontaneously”, just as neurologists look for imbalance in Romberg's test by asking the patients to close their eyes.

Clinicians can, for example, explore psychomotricity by examining the reaction of the subject to stimuli (verbal request, exaggerated gesture), making it possible to observe the tendency to initiate a bizarre automatic response (echolalia/ echomimia /echopraxia) or increased response latency, or even automatic resistance to verbal or non-verbal requests (50). For subjects with spontaneous fluid speech, they can propose more restrictive conditions, for example, by asking the subject to explain the differences between pairs of words relating to tangible (box/basket) or abstract (error/lie) entities, to reveal small logical or conceptual difficulties (51).

The *second principle* is to characterize “disease individuality,” “describing the subject observed and what distinguishes that subject individually, rather than describing the phenomena common to this and other subjects according to existing classifications” (49).

Finding differences between subjects with ASD of different etiologies obviously requires us to look for their singularities rather than the features they have in common. The information collected by the ADI-R or the Autism Diagnostic Observation Schedule (ADOS) (52), which seek common manifestations of ASD, should not, therefore, *initially* be taken into account.

Alongside the most salient symptoms, the identification of “negative findings” defined as “the absence of certain findings under conditions in which they should necessarily occur,” can also be discriminating in the differential clinical approach. Such findings may correspond not only to developmental delays (e.g., lack of protodeclarative pointing at 18 months), with a reference framework of normal development, but also to the absence of certain typical “autistic traits” in ASD subjects of a given etiology relative to prototypical autism (9). For example, deviant language development (delayed echolalia, pronoun reversal) or atypical visual exploratory behavior is regularly absent from the “autistic traits” of subjects with *SHANK3* abnormalities (53).

Finally, close longitudinal observation to assess the temporal dynamics of the natural course also contributes to the characterization of “disease individuality” (47, 48).

The *third principle* is “never to separate a finding from the condition from which it arises, or from the circumstances that precede or follow its occurrence” (47). Detached from their context, the signs or symptoms lose their significance; it is the context that allows its clinical value to be assessed (54). “It is not enough to note the odd and extraordinary words pronounced by the insane patient, and the eccentric and muddled actions he committed; one must, above all, assess and carefully analyze the internal psychic state that gives birth to these words and actions” (47, 49). “When faced with an agitated patient, it is therefore important to search carefully for the cause of such agitation, to determine whether it is automatic and muscular, or voluntary, driven by an idea” (47). Returning to the clinical case of “An 8-year-old boy with “odd behavior”' taking the context into account makes it possible to understand the differences in the diagnostic process. This boy seemed to be very comfortable in interactions involving a mediated activity (class presentations, speaking through a microphone) and when he took the initiative for the exchange, whereas he seemed more troubled when he was solicited or had to interact directly with his peers (30).

Understanding ASD requires symbiosis between the life sciences and the sciences of the mind, two disciplines that differ in their objectives, methods, and experimental design. They often see each other as rivals, each tending to claim a monopoly on explanation or understanding, whereas here, in the specific case of ASD caused by a rare variants, it is precisely their differences in approach that make it possible to validate results reciprocally, demonstrating the pathogenicity of the variant and the specificity of the psychopathological profile.

Studies of subjects carrying rare mutations provide an opportunity to understand the physiological and developmental functions of the genes concerned, but, above all, they also provide unique access to the psychopathological impact of each mutation.

Indeed, the grouping together of subjects carrying the same rare mutation makes it possible, by taking into account both chronological and development age, to perceive the underlying psychopathological prototype beyond the background noise of temperament, individual history, and sociocultural influences, provided that we do not get cling to our stereotypes and that we systematically verify our perceptions. An awareness of these psychopathological profiles will open up new perspectives for research, diagnostic genomic testing and clinical practice.

In the field of research, this will make it possible to examine the functional relationships linking psychopathological phenotypes and physiological dysfunctions through *in vivo* studies (functional imaging, electrophysiology, animal models, etc.) or *in vitro* studies (cell models based on cells directly derived from patients, etc.). In diagnostic genomic testing, a knowledge of psychopathological characteristics will facilitate clinical interpretation, by biologists, of genetic variants that have not been encountered before. In the clinical setting, disentangling psychopathological profiles on the autism spectrum will improve our understanding of the mental state of patients, making it possible to provide more personalized treatment.

Finally, although monogenic forms represent a small fraction of ASD cases, this change in perspective concerning the way we view the signs and symptoms of ASD with rare etiologies may provide insight to improve clinical judgments for more prevalent idiopathic forms of ASD, and may facilitate the identification of mutations in the subjects described by Asperger, Newson, or Wing.

## Data Availability Statement

The original contributions presented in the study are included in the article. Further inquiries can be directed to the corresponding author.

## Author Contributions

The author confirms being the sole contributor of this work and has approved it for publication.

## Conflict of Interest

The author declares that the research was conducted in the absence of any commercial or financial relationships that could be construed as a potential conflict of interest.

## Publisher's Note

All claims expressed in this article are solely those of the authors and do not necessarily represent those of their affiliated organizations, or those of the publisher, the editors and the reviewers. Any product that may be evaluated in this article, or claim that may be made by its manufacturer, is not guaranteed or endorsed by the publisher.
